# Mobile Money, Smallholder Farmers, and Household Welfare in Kenya

**DOI:** 10.1371/journal.pone.0109804

**Published:** 2014-10-06

**Authors:** Enoch M. Kikulwe, Elisabeth Fischer, Matin Qaim

**Affiliations:** Department of Agricultural Economics and Rural Development, Georg-August-University of Goettingen, Goettingen, Germany; Monash University, Australia

## Abstract

The use of mobile phones has increased rapidly in many developing countries, including in rural areas. Besides reducing the costs of communication and improving access to information, mobile phones are an enabling technology for other innovations. One important example are mobile phone based money transfers, which could be very relevant for the rural poor, who are often underserved by the formal banking system. We analyze impacts of mobile money technology on the welfare of smallholder farm households in Kenya. Using panel survey data and regression models we show that mobile money use has a positive impact on household income. One important pathway is through remittances received from relatives and friends. Such remittances contribute to income directly, but they also help to reduce risk and liquidity constraints, thus promoting agricultural commercialization. Mobile money users apply more purchased farm inputs, market a larger proportion of their output, and have higher profits than non-users of this technology. These results suggest that mobile money can help to overcome some of the important smallholder market access constraints that obstruct rural development and poverty reduction.

## Introduction

During the last decade, mobile phone technologies have spread rapidly in many developing countries [1], [2]. Several studies showed that mobile phones can cause significant benefits for rural households through improved access to information, lower marketing costs, and thus higher profits and incomes [3]–[7]. In addition to such direct effects, mobile phones are an enabling technology for other innovations. One important example are mobile phone based money transfers, which could be very relevant for rural households that are often underserved by the formal banking system. So far, little is known about the impact of mobile money on the livelihoods of the rural poor.

Mobile money services were introduced by private telecommunication providers in several countries of Africa, Asia, and Latin America [8]. The general idea is to enable cheap and reliable money transfers between people that have access to a mobile phone. This is especially relevant for sending and receiving remittances, which is much more expensive and sometimes risky through traditional formal and informal mechanisms [9], [10]. In addition, mobile money facilitates transfers between business partners [11]–[13], reducing transaction costs and promoting market exchange. Finally, mobile money services provide relatively secure opportunities for saving, even in remote rural areas [14], [15].

While these potential effects of mobile money were identified in principle, there are only a few studies that have analyzed impacts on household welfare empirically. Some studies were initiated by telecommunication providers to demonstrate the viability of their business model; results are not always representative [16]. Most existing research on this topic uses qualitative approaches [9], [17], [18]. One exception is Suri et al. [19], who used household panel data to analyze the impact on risk sharing in Kenya; they showed that mobile money users could smooth their consumption due to remittances received in times of economic shocks. In another quantitative study, Mbiti and Weil [12] used aggregate data to show that mobile money use has positive effects on different economic indicators, including employment. Both studies did not analyze the impact on household income. Nor did they investigate what mobile money use could mean for agricultural production, the main economic activity of the rural poor. The only study that analyzed the effects of mobile money on agricultural production and income is Kirui et al. [20]; they used cross-section data from farm households in Kenya to estimate impacts on agricultural income.

We add to this literature and analyze welfare effects of mobile money building on panel data from smallholder farm households. Panel data are more suitable to control for possible selection bias in impact assessment. We examine potential impact pathways of mobile money in terms of remittances received, transactions in input and output markets, and farm profits. Moreover, we analyze effects on total household income, including farm and non-farm sources. Given diversified income sources in the small farm sector, total household income is a more comprehensive welfare measure than agricultural income. Our analysis concentrates on farm households in Kenya, where mobile money services have spread rapidly in recent years [13], [20]. In 2007, Safaricom, Kenya’s largest mobile service provider, launched a mobile money program called M-Pesa (the letter “M” refers to mobile, and Pesa means money in Swahili). Since 2009, a few other companies have launched similar programs in Kenya under different names.

The rest of this article is structured as follows. In the next section, we develop a conceptual framework, discussing how mobile money can affect the income of farm households that otherwise have limited access to formal financial services. This is followed by a description of the survey data and the empirical strategy to estimate impacts. Subsequently, the estimation results are presented and discussed.

## Conceptual Framework

**Figure 1:**
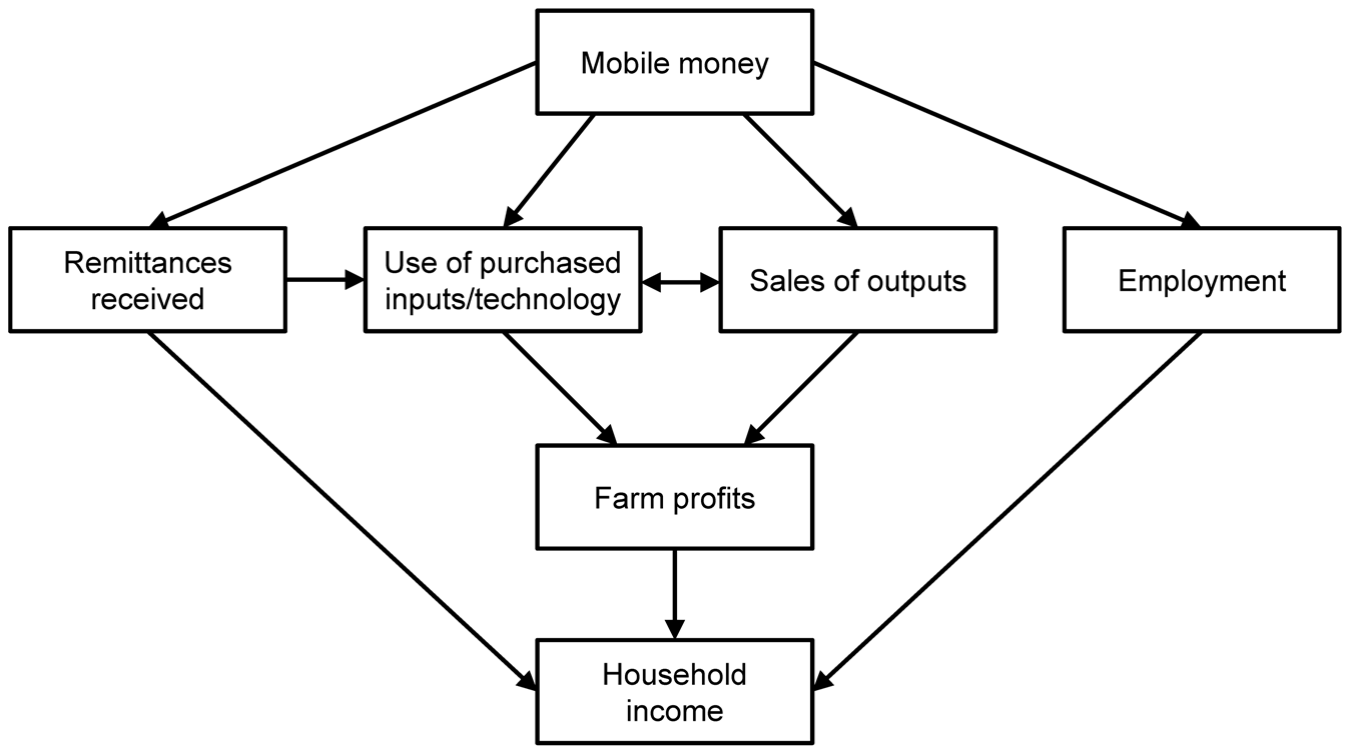
Impact pathways of mobile money.

Availability and use of mobile money services can affect household income through multiple pathways, as shown in Figure 1. The effects could be especially important for poor people in rural areas for whom traditional banks and related financial services are often inaccessible. The first possible pathway is through remittances received, often from relatives and friends who migrated to urban areas. Many studies show that remittances constitute an important component of rural household income and are used for different productive and consumptive purposes [21]–[23]. Without access to mobile money services, remittances can be sent through banks. However, the financial system is often underdeveloped in rural areas, so that bank services are not available everywhere [24]. Moreover, hefty fees are often charged, especially when the recipient does not have a bank account. Alternatively, cash is sometimes sent through persons traveling to the destination, such as bus or truck drivers, but such informal mechanisms are also associated with high transaction costs and they are less safe. Mobile money services reduce the transaction costs considerably, because money can be transferred by sending a simple text message to the recipient’s mobile phone. Due to its cheapness and reliability, mobile money is now the main avenue for sending and receiving remittances in Kenya [9], [15], [25]. While the risk of misappropriation cannot be ruled out completely, mobile money services are much safer than most informal means of cash transfer. Studies show that rural households are more likely to receive remittances from their distant relatives and friends through mobile money technology. Likewise, urban households with relatives in rural areas were found to use mobile money services more frequently. Interestingly, for M-Pesa in particular the senders are mostly men in urban areas, while the recipients are mainly women in rural areas [9]. Similar effects of mobile money services on remittances were also revealed in other countries, such as Uganda, Tanzania, and the Philippines [11], [26], [27].

A second possible pathway of how mobile money can affect the income of farm households is through more intensive use of purchased agricultural inputs and technologies, including fertilizers, pesticides, and hired labor, among others. Market participation by smallholder farmers is often relatively low, due to high transaction costs, liquidity constraints, and risk aversion [28]–[31]. Mobile money is unlikely to solve all these constraints, but it may improve the situation. For instance, inputs may be purchased but paid at a later date without the farmer having to go to the input shop again. Similarly, wages for hired farm laborers can be paid more easily and flexibly, without having to keep large amounts of cash. Savings and remittances received may also help to ease liquidity constraints and risk [10], [14]. Suri et al. [19] showed that remittances sent or received through mobile money technology tend to reduce the impact of negative economic shocks, thus providing a form of insurance. Mbiti and Weil [12] found that mobile money services decreased the propensity to use informal savings and insurance mechanisms. Such informal savings and insurance mechanisms were shown to affect investment behavior and reduce economic efficiency in some situations [32], [33]. Hence, access to mobile money services is expected to increase farmers’ willingness and ability to invest in agricultural inputs, which may increase productivity, profits, and thus household income.

A third and related pathway is through higher degrees of commercialization on the output side. Higher input use and productivity through mobile money may contribute to more marketable surplus and thus higher farm profits and household incomes. The relevance of these pathways will be tested empirically below for the example of farm households in Kenya.

There are also other possible impact pathways. Access to mobile money may facilitate farmers’ integration into high-value supply chains. For instance, a recent study in Kenya showed that sales to supermarkets are often associated with payments that are delayed by several days [34]. In such situations, a cheap and reliable system of money transfer could reduce market entry barriers for smallholders. In addition, there may be positive employment effects. Given that mobile money use is associated with higher economic activity, labor demand is likely to increase, which could improve farmers’ off-farm employment opportunities. Results by Mbiti and Weil [12] suggest that mobile money has contributed to increased employment in Kenya. We do not analyze such other pathways explicitly due to data limitations. Yet it should be kept in mind that additional mechanisms may be at work when interpreting the observed household welfare effects.

## Materials and Methods

### Ethics statement

Our study builds on data from a socioeconomic survey of farm households in Kenya. The institutional review board of the University of Goettingen only reviews clinical research; our study cannot be classified as clinical research. We consulted with the Head of the Research Department of the University of Goettingen, who confirmed that there is no institutional review board at our University that would require a review of such survey-based socioeconomic research.

Households that participated in the face-to-face interviews were selected randomly (see sampling details below). Interviews were carried out in the local language by trained enumerators, who were supervised by the researchers. Participation was voluntary. Prior to starting each interview, the study objectives were explained to the respondents. It was also clarified that the data collected would be treated confidentially, analyzed anonymously, and be used for research purposes only. Based on this, the interviewees were asked for their verbal informed consent to participate. We decided not to ask for written consent, because the interviews were not associated with any risk for participants. Furthermore, many of the sample farmers had relatively low educational backgrounds and were not used to formal paperwork.

### Household panel survey

The survey of farm households was conducted in Central and Eastern Provinces of Kenya (after the constitutional change in 2013, provinces do not exist as administrative units anymore). As this was part of a project to analyze socioeconomic conditions and innovations in the Kenyan banana sector, the sampling framework focused on the main banana-growing areas. Within Central and Eastern Provinces, the districts of Meru, Embu, Kirinyaga, Kiambu, Murang’a, and Thika were selected (districts are now referred to as counties in Kenya). In each district, banana-growing villages (sublocations) were purposively sampled. Within the selected villages, households were randomly sampled based on complete household lists. The first round of data collection was carried out at the end of 2009, referring to production and income in 2009. A second round of the survey with the same households was implemented at the end of 2010, referring to production and income in 2010. The balanced panel comprises 640 observations from 320 households that were interviewed in both survey rounds (Data S1).

Sample households are diversified smallholders, most of them with farm sizes of less than 5 acres. All of the households in the sample grow banana for home consumption and sales in local markets. Yield levels are relatively low due to low input use and severe problems with pests and diseases. Recently, different organizations – including the Kenya Agricultural Research Institute (KARI), Africa Harvest, and others – have implemented projects to improve the productivity of banana production through the promotion of tissue culture planting material and related technical information [35], [36]. In addition to banana, sample farms grow maize and different horticultural crops. Many also have some livestock activities such as raising chicken and small ruminants, and some grow cash crops such as coffee on a small scale. The sample is representative of smallholder banana growers in Central and Eastern Kenya.

Using a structured questionnaire, we collected data on household human capital and demographic characteristics, banana production and other farm enterprises, as well as off-farm economic activities. One special section of the questionnaire focused on mobile phone ownership and use of mobile money services. Sample descriptive statistics are provided below.

### Regression models

The main focus of this study is to analyze impacts of mobile money use among smallholder farm households. As mentioned, mobile money services have spread rapidly in Kenya during the last few years. Nonetheless, not all households use mobile money, so that a first question of interest is as to what factors influence the adoption of this innovation. This is analyzed with a probit model:
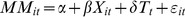
(1)where the dependent variable 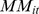 is a dummy that takes a value of one if household *i* has used mobile money services in year *t*, and zero otherwise. 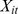 is a vector of farm, household, and contextual characteristics that may influence the decision to use mobile money; some of these characteristics may vary over time, while others are time-invariant. 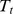 is a year dummy to control for time fixed effects, and 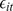 is a random error term with a standardized normal distribution.

To analyze impacts we use panel models of the following type:
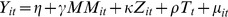
(2)where 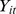 is the continuous outcome variable of interest (e.g., income, remittances received). In these models, we use 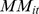 as treatment dummy. As before, 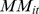 takes a value of one if household *i* has used mobile money services in year *t*. Hence, 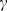 is the treatment effect of mobile money use on the outcome variable. 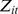 is a vector of relevant covariates, which may include both time-variant and time-invariant factors. Again, we include a year dummy 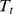 to control for time fixed effects. 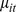 is the random error, which includes unobserved individual effects that may be constant or also time-variant.

Equation (2) can be estimated with a random effects (RE) estimator. However, 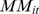 may potentially be correlated with the error term due to unobserved heterogeneity between mobile money users and non-users. Such heterogeneity is likely, as households self-select into the group of users. If not controlled for, this could lead to selection bias in the estimated treatment effects. A common way to reduce the issue of selection bias is to use a household fixed effects (FE) estimator [37]. The FE estimator builds on a differencing approach within households, so that all time-invariant factors cancel out, even when they are unobserved. Efficient FE estimates require within-group variability with respect to the treatment variable. That is, there needs to be a sufficient number of households who used mobile money services in one year of the survey, but not in the other year. Such variability is given in our data, because we surveyed during a time when mobile money services were spreading fast in rural Kenya. We estimate all models with both RE and FE estimators, and use a Hausman test to compare results [37]. However, recent studies showed that a significant Hausman test statistic is neither a necessary nor a sufficient condition to detect unobserved heterogeneity [38]. Hence, we will show both results, yet preferring the FE estimates for interpretation of the mobile money treatment effects.

All outcome variables are continuous, but some of them are censored at zero. For instance, households that did not receive any transfers from relatives or friends in a particular year reported zero remittances. Using the common linear specification for models with a censored dependent variable may potentially lead to biased estimates [37]. Hence, for outcome variables where this is relevant we additionally use a Tobit estimator. As Tobit panel models cannot be estimated with household fixed effects, we only show the RE Tobit estimates for comparison. We carry out additional robustness checks using instrumental variable (IV) and inverse probability weighting (IPW) estimators, both of which are common tools in the impact assessment literature [39].

### Dependent and independent variables

For the impact models, the main outcome variable of interest is total household income, which is calculated as the sum of all net earnings from on-farm and off-farm sources. In the survey, we collected data on output quantities, output values, and input costs for all crop and livestock enterprises over a 12-months recall period. For crops, we differentiated by season (long rains and short rains) to improve data accuracy. Revenues from off-farm sources, including self-employed non-agricultural activities, and related costs were also elicited for a 12-months period, covering activities of all household members. A 12-months recall period is relatively long, which may lead to some inaccuracy in the income data. However, for the calculation of annual incomes a shorter recall period would have led to even higher inaccuracy, because of significant seasonal differences.

In the calculation of total household income, remittances are included as an off-farm income source. In a separate impact model, we use remittances as outcome variable, including all transfers from relatives and friends not residing in the household. The treatment variable of interest is mobile money, which is captured as a dummy that takes a value of one if mobile money services were used in the particular year and zero otherwise.

To estimate the impact of mobile money on the use of agricultural inputs and output sales we concentrate on the banana crop. Mobile money can also affect other agricultural enterprises, but there are two particular reasons why we decided to take this partial perspective. First, concentrating on one crop allowed us to collect more detailed and disaggregated data on the use of specific inputs. Second, banana is a typical semi-subsistence crop in Kenya, which is often cultivated with relatively low amounts of purchased inputs [35]. Thus, the effects of mobile money services may be more pronounced than for typical cash crops that are grown with higher input intensities anyway. We concentrate on hired labor, purchased organic and mineral fertilizer, and chemical pesticides used in banana production. The use of each of these inputs is expressed in monetary terms per acre and used as dependent variable in separate model specifications.

To assess the impact of mobile money on output commercialization, we use the proportion of bananas sold in the market relative to total banana production as dependent variable. Most farmers sell their bananas as bunches at the farm gate to local traders. Some of the farmers are organized in groups, selling bananas during collective marketing days to wholesalers coming to the region from Nairobi and other urban centers [40]. To estimate potential mobile money impacts on profits, we use banana profit per acre as dependent variable; this is calculated as the market value of output (including home-consumed quantities valued at market prices) minus the cost of all purchased inputs.

As covariates in the different models, we include farm and household characteristics such as farm size (land owned), household size, as well as gender, age, and education of the household head. These variables may influence income, agricultural decisions, and also the decision whether or not to use mobile money services. In addition, we include contextual variables, such as the distance of the household to markets and roads. Agro-ecological conditions are captured through a ‘high-potential area’ dummy, which takes a value of one for regions with more fertile soils and higher amounts of rainfall, and zero otherwise. High-potential areas especially comprise the slopes of Mount Kenya, including the districts of Embu, Meru, and the northern half of Kirinyaga. Finally, for the probit model to explain the use of mobile money services, we include a variable measuring the percentage of households using mobile phones at the village level to capture neighborhood effects. It is expected that a wider use of mobile phones in the community increases the likelihood of individual households to also use mobile phones and related services.

## Results and Discussion

### Patterns of mobile money use

Table 1 shows how mobile phone and mobile money use developed over the 2009–2010 period covered by the panel survey. In 2010, 93% of all sample households owned at least one mobile phone, which was up from 86% in 2009. The difference between the two survey rounds was much stronger for the use of mobile money services, which increased from 60% in 2009 to 91% in 2010. We also asked farmers for the distance to the nearest shop offering mobile money services, such as withdrawing or depositing money on the mobile account. In 2010, the average distance was only 2 km, which underlines the wide coverage of these services in rural areas.

**Table 1:** Use of mobile phones and mobile money among sample households.

	2009		2010	
Variable	Mean	Std. Dev.	Mean	Std. Dev.
Proportion of mobile phone owners	0.86	0.35	0.93***	0.26
Proportion of mobile money users	0.60	0.49	0.91***	0.28
Years owning a mobile phone	3.78	2.92	4.71***	3.02
Years using mobile money	0.94	0.94	1.85***	1.07

***mean value between 2009 and 2010 is significantly different at the 1% level.

**Figure 2:**
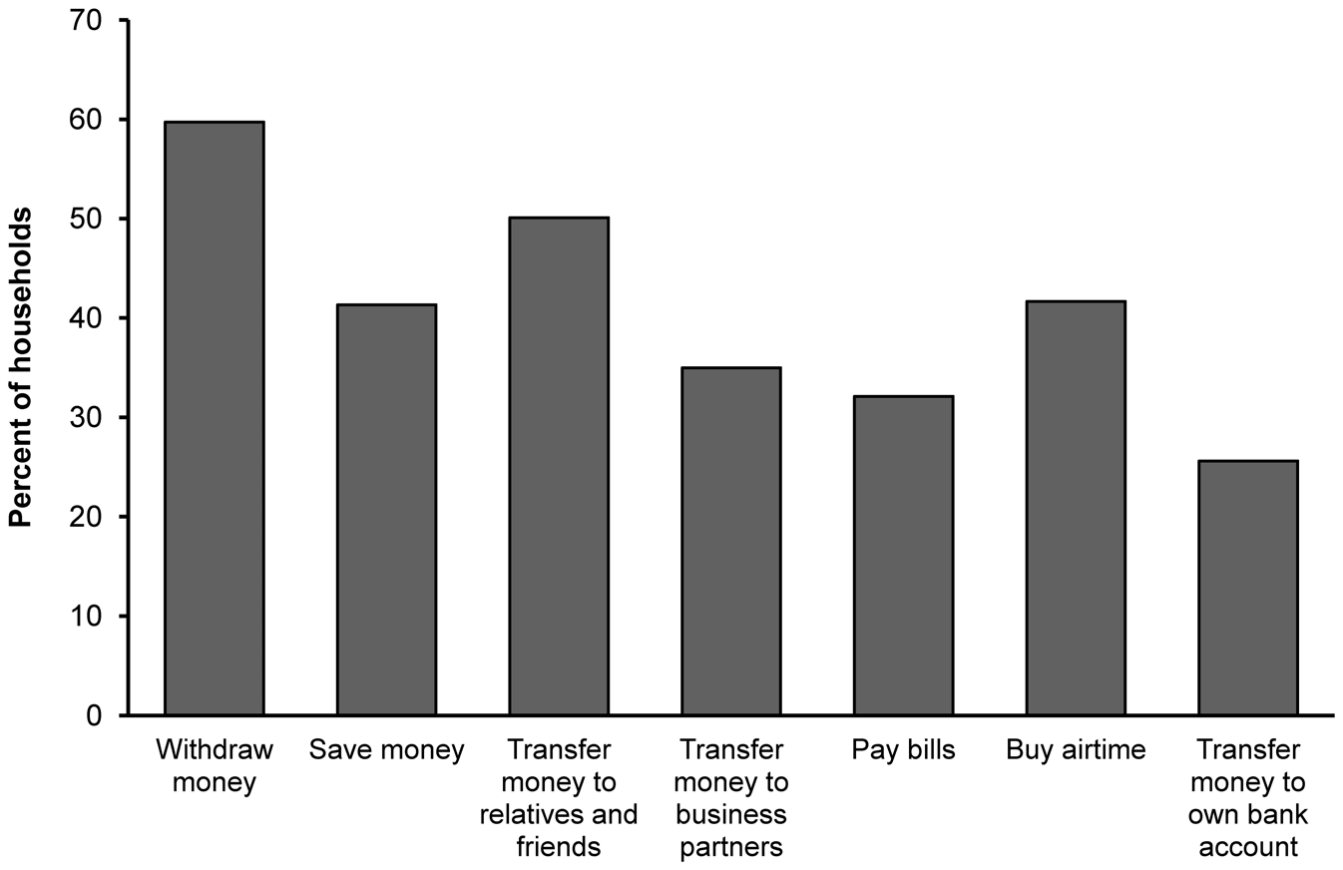
Types of activities performed with mobile money among sample households.

Figure 2 shows for what concrete activities sample households used mobile money services in 2010. Around 60% of the households stated that they withdraw money from their mobile account, which may be money from remittances, payments by traders, or also from previous own saving deposits. Over 40% of the households stated that they use their mobile money account as a savings tool. The households do not only receive remittances; about 50% stated that they also transferred money to other relatives and friends. Thirty-five percent used mobile services to transfer money to business partners, such as input dealers or farm laborers, while 32% stated that they transferred mobile money to pay for regular water or electricity bills. More than 40% of the households use mobile money to buy airtime for their mobile phone. Interestingly, about 27% also used mobile money services as a means of transferring money to their formal bank account, which is possible when the mobile provider has a special agreement with the respective bank.

While the concrete numbers vary, the overall patterns of mobile money services observed in our sample are similar to those reported in earlier research in Kenya [12], [15]. Especially the payment of bills and the transfer of money to business partners through mobile money services seem to have increased over time. It is also worth mentioning that most farm households in our sample use mobile money for different activities: 93% of the adopters used mobile money for more than one activity, 80% for more than two activities, and 60% for more than three activities.

### Descriptive statistics

Table 2 shows descriptive statistics for the variables used in the regression models. For comparison, we differentiate between users and non-users of mobile money services. The upper part of the table shows the outcome variables for the impact assessment models. The columns for the pooled sample, which covers both survey rounds, reveal that mobile money users had significantly higher household incomes than non-users. Users had an annual mean income of 283 thousand Kenyan shillings (Ksh), which is equivalent to 3435 US$ per household, or around 735 US$ per capita. Non-users had an annual income of 153 thousand Ksh, equivalent to 1854 US$ per household, or 458 US$ per capita. Users of mobile money also had higher profits from banana production and sold a larger proportion of their harvest. As expected, they used significantly higher amounts of purchased inputs per acre of banana production.

**Table 2:** Descriptive statistics for variables used in regression models.

	Pooled sample				2009				2010			
Variable	MM users	SD	Non-users	SD	MM users	SD	Non-users	SD	MM users	SD	Non-users	SD
*Outcome variables*												
Household income (000 Ksh)	283.35***	228.59	152.98	142.70	250.17***	243.14	138.09	116.30	305.05*	216.23	221.56	218.18
Remittances (000 Ksh)	10.91	48.92	6.67	21.71	19.52**	74.00	6.27	22.05	5.28	17.55	8.46	20.36
Banana profit (000 Ksh/acre)	110.94**	124.03	85.65	99.71	92.51*	94.87	76.05	68.12	122.99	138.69	129.87	181.50
Proportion of banana sales	0.69***	0.38	0.56	0.27	0.63***	0.25	0.55	0.27	0.74	0.43	0.61	0.27
Hired labor (000 Ksh/acre)	6.36***	12.31	2.95	13.31	2.37	5.47	1.51	4.28	8.97	14.64	9.60	29.69
Organic fertilizer (000 Ksh/acre)	3.54***	8.34	0.94	3.84	1.63	6.69	0.73	4.01	4.78*	9.06	1.90	2.84
Mineral fertilizer (000 Ksh/acre)	4.46***	8.29	1.23	5.42	0.79	3.68	0.98	5.85	6.47**	9.51	2.39	2.45
Pesticides (000 Ksh/acre)	2.08***	4.71	0.33	1.47	0.28	1.36	0.24	1.49	3.26**	5.66	0.71	1.31
*Explanatory variables*												
Land owned (acres)	3.43	2.96	3.06	3.09	3.50	2.86	3.11	3.18	3.39	3.03	2.86	2.67
Age of household head (years)	58.14	13.30	61.04	14.45	58.45	13.45	59.45	13.97	57.94***	13.22	68.36	14.63
Education (years)	8.99	3.88	6.78	4.10	9.21***	3.95	7.31	3.93	8.84***	3.83	4.30	4.00
Household size (members)	4.67	2.07	4.05	2.07	4.75**	1.97	4.29	2.07	4.63***	2.13	2.93	1.74
Male household head (dummy)	0.84**	0.36	0.77	0.42	0.85	0.36	0.79	0.41	0.84**	0.37	0.68	0.48
Distance to banana market (km)	4.26	3.59	4.24	3.62	4.28	3.62	4.21	3.57	4.24	3.57	4.35	3.90
Distance to all-weather road (km)	3.62	3.79	3.50	3.84	3.63	3.74	3.53	3.92	3.62	3.64	3.32	3.51
High-potential area (dummy)	0.55	0.50	0.56	0.50	0.54	0.50	0.58	0.50	0.56	0.50	0.50	0.51

Notes: MM, mobile money; SD, standard deviation.

*,**,***mean value between MM users and non-users in the same period is significantly different at the 10%, 5%, and 1% level, respectively.

The disaggregation by survey round reveals relatively large differences for most variables between 2009 and 2010. The reason is that 2009 was a year with below average amounts of rainfall in Central and Eastern Kenya. Hence, input use, profits, and incomes were lower in 2009 than in 2010, when rainfall was more favorable. For remittances, the pattern is different: especially for users of mobile money, remittances received were significantly higher in 2009 than in 2010. Remittances received by smallholder farm households are often found to be higher in years with below average agricultural incomes.

The lower part of Table 2 shows descriptive statistics of the explanatory variables used in the regression models. Most of the mean values are not significantly different between users and non-users of mobile money services. However, a few differences can be observed. Households that use mobile money are more likely to be male-headed. The disaggregated data for the two survey rounds also shows that larger households and those with better educated household heads are more likely to use mobile money.

### Determinants of mobile money use

Estimation results from the probit model explained in equation (1) above are shown in Table 3. Column (1) represents the main model to explain mobile money use. Several variables turn out to be significant. While age does not play a significant role, the education level of the household head affects mobile money use in a positive way. Each additional year of schooling increases the probability of using mobile money services by 1.7 percentage points. Household size also plays a significant role; households with more members are more likely to use mobile money. Further, the results suggest that wealth, proxied by farm size, influences the household decision. Each additional acre of land owned increases the probability of mobile money use by 2.3 percentage points. The negative square term indicates that this effect diminishes at larger farm sizes. The market access variables, including distance to the nearest banana market and to road infrastructure, are not significant. Nor do agro-ecological conditions – captured by the high-potential area variable – seem to play a role. These are welcome findings, because they indicate that households in remoter and less favorable areas are also able to use mobile money services. As supposed, due to the wide coverage and network of shops set up by private telecom providers in rural areas, mobile applications can help to overcome some of the typical market access constraints of smallholder farm households.

**Table 3:** Determinants of mobile money and mobile phone use (probit model estimates).

	(1)	(2)	(3)
Variable	Mobile money	Mobile money	Mobile phone
Age of household head	0.008 (0.007)	−0.005 (0.006)	0.014*** (0.004)
Age squared	−6.8E-05 (5.8E-05)	5.6E-05 (5.5E-05)	−1.3E-04*** (3.5E-05)
Education of household head	0.017*** (0.004)	0.010*** (0.004)	0.011*** (0.003)
Male household head	0.027 (0.037)	0.015 (0.030)	0.010 (0.026)
Household size	0.017** (0.008)	0.008 (0.006)	0.013*** (0.005)
Land owned	0.023** (0.010)	0.008 (0.008)	0.022*** (0.008)
Land squared	−0.001** (4.6E-04)	−0.001* (3.7E-04)	−6.1E-04* (3.6E-04)
Distance to banana market	0.001 (0.004)	0.003 (0.004)	−0.001 (0.003)
Distance to all-weather road	0.003 (0.004)	1.7E-04 (0.003)	0.002 (0.003)
High-potential area	−0.008 (0.029)	0.011 (0.025)	−0.022 (0.022)
Percentage of village households with mobile phone	0.008*** (0.001)	0.005*** (0.001)	0.005*** (0.001)
2010 dummy	0.317*** (0.028)	0.267*** (0.025)	0.076*** (0.021)
Mobile phone ownership		0.470*** (0.061)	
*Model statistics*			
Pseudo R^2^	0.282	0.448	0.292
Wald χ^2^	139.49***	133.80***	93.93***
Number of observations	640	640	640

Notes: Marginal effects are shown with standard errors in parentheses.

*,**,***significant at the 10%, 5%, and 1% level, respectively.

The percentage of households owning a mobile phone at the village level has a positive effect on mobile money use of the individual household. As we control for other location related variables, we conclude that neighborhood effects are significant. A large percentage of households with a mobile phone indicates that many in the community are likely to be familiar with mobile applications. Recent research has shown that social networks and related knowledge transfer can play an important role for innovation adoption [41]. The 2010 year dummy is also highly significant, showing that – independent of other variables included in the model – the use of mobile money services has increased rapidly in Kenya. As mentioned, in 2010 already 91% of the sample households used mobile money services.

Individual ownership of a mobile phone is a precondition for using mobile money services. Therefore, we include ownership of a mobile phone as an additional explanatory variable in the probit model shown in column (2) of Table 3. As expected, this variable has a highly significant effect; ownership of a mobile phone increases the probability of mobile money use by 47 percentage points. At the same time, some of the other explanatory variables lose their significance, which is due to their correlation with mobile phone ownership. Several of the characteristics that determine mobile money use also determine mobile phone ownership, as is demonstrated in column (3) of Table 3.

### Impact of mobile money on household income

The factors influencing household income are presented in Table 4. These estimates build on equation (2), using total annual household income as dependent variable. The results in column (1) are based on the FE estimator, while column (2) shows results with the RE estimator. As explained, for interpretation of the mobile money impact we prefer the FE results, as these account for unobserved heterogeneity between mobile money users and non-users. Results in column (1) suggest that mobile money use is associated with significantly higher income. The estimated treatment effect of 61.5 thousand Ksh (745 US$) implies an income increase of 40% relative to the mean income of non-users of mobile money. The 2010 dummy coefficient is also large and significant, implying that household incomes were higher in 2010 than in 2009. This is expected, because 2010 was a year with more favorable rainfall.

**Table 4:** Determinants of household income.

	(1)	(2)	(3)	(4)
Variable	FE	RE	FE	FE
Mobile money (dummy)	61.470* (32.704)	70.694*** (21.312)	18.990** (8.899)	
Number of mobile money users in household				32.021* (17.866)
2010 dummy	73.343*** (18.373)	71.458*** (16.516)	21.115*** (4.999)	76.033*** (17.838)
Age of household head		0.540 (0.732)		
Education of household head		9.408*** (2.510)		
Male household head		−13.430 (23.772)		
Household size		11.729*** (4.141)		
Land owned		6.648** (3.034)		
Distance to banana market		0.326 (0.514)		
Distance to all-weather road		4.090* (2.291)		
High-potential area		0.721 (17.533)		
Intercept	168.307*** (22.283)	−7.290 (63.957)	40.390*** (6.063)	168.673*** (22.943)
*Model statistics*				
Wald χ^2^		96.93***		
*F* value	20.38***		23.76***	20.20***
Hausman test, χ^2^	0.37			

Notes: Estimates are based on balanced panel regressions with 640 observations and 320 groups. The dependent variable in columns (1), (2), and (4) is total household income. The dependent variable in column (3) is per capita income. Incomes are measured in thousand Ksh/year. Coefficient estimates can be interpreted as marginal effects; standard errors are shown in parentheses. FE, fixed effects; RE, random effects.

*,**,***significant at the 10%, 5%, and 1% level, respectively.

In the FE model, all other covariates were dropped, as these are time-invariant (though time-variant, age was also dropped, because of the close correlation with the 2010 dummy). Nonetheless, it is interesting to see what role these other factors play for household income, which is shown in the RE results in column (2) of Table 4. Education of the household head has a positive effect on income; each additional year of schooling increases annual income by 9400 Ksh. Likewise farm size and household size have a positive effect on income. The latter should not surprise because the dependent variable is total income per household, not per capita. Somewhat unexpected is the positive effect for distance to the next all-weather road, which is significant at the 10% level. Probably distance alone is not a very comprehensive measure of market access constraints [30].

Column (3) of Table 4 shows the same FE specification as column (1), but this time using per capita income as dependent variable. The results confirm that mobile money is also associated with significantly higher per capita income. In column (4), we use household income as dependent variable, but we define the treatment variable in a different way. Instead of a dummy for mobile money use, we use the number of mobile money users in the household as a measure of treatment intensity. In 66% of the sample households with mobile money, more than one member, and in 19% of the households more than two members, use mobile money services. The estimation results in Table 4 reveal a significant effect on household income also with this changed specification of the treatment variable.

### Impact of mobile money on remittances received

Table 5 presents results for the remittances models, again with FE and RE specifications shown in columns (1) and (2), respectively. Mobile money use is associated with significantly higher remittances received. The estimated treatment effect of 12.7 thousand Ksh (154 US$) per year implies an increase of 66% compared to the mean remittances received by non-users of mobile money. The negative and significant coefficient of the 2010 dummy indicates that remittances received were lower in 2010 than in 2009. Column (2) shows that larger households and those with older household heads received higher remittances on average.

**Table 5:** Determinants of remittances received.

	(1)	(2)	(3)
Variable	FE	RE	Tobit RE
Mobile money	12.697** (6.461)	12.435*** (4.378)	23.722** (11.300)
2010 dummy	−12.625*** (3.630)	−12.543*** (3.303)	−33.947*** (9.573)
Age of household head		0.616*** (0.154)	2.831*** (0.423)
Education of household head		−0.390 (0.530)	−0.379 (1.256)
Male household head		−15.984 (13.603)	−33.102*** (11.568)
Household size		1.819* (0.932)	1.917 (2.178)
Land owned		−0.191 (0.641)	−0.566 (1.470)
Distance to banana market		−0.586 (0.518)	−0.496 (1.288)
Distance to all-weather road		−0.249 (0.490)	−2.468* (1.361)
High-potential area		−2.663 (3.737)	−5.249 (9.111)
Intercept	6.661 (4.402)	−15.984 (13.603)	−159.158*** (36.511)
*Model statistics*			
Wald χ^2^		55.66***	93.60***
*F* value	6.05***		
Hausman test, χ^2^	0.00		
Log likelihood			−2545.30

Notes: Estimates are based on balanced panel regressions with 640 observations and 320 groups. The dependent variable in all models is remittances received per household (thousand Ksh/year). Coefficient estimates can be interpreted as marginal effects; standard errors are shown in parentheses. FE, fixed effects; RE, random effects.

*,**,***significant at the 10%, 5%, and 1% level, respectively.

Since not all sample households had received remittances, the dependent variable is censored at zero. We therefore additionally estimated a RE Tobit model, results of which are shown in column (3) of Table 5. The signs and significance levels of the main variables of interest remain unaffected, but most of the coefficients increase in magnitude. Hence, while the exact numerical results should be interpreted with some caution, this additional model further underlines the significance of the mobile money treatment effect. The Tobit results also produce a few significant coefficients that were insignificant in the linear specifications. For instance, male-headed households received significantly lower remittances than female-headed households.

### Impact of mobile money on input use

We hypothesized above that mobile money services may increase the use of agricultural inputs through various channels. We test this hypothesis for hired labor, organic and mineral fertilizers, and chemical pesticides, which are used by many sample farmers in their banana crop. The estimation results are shown in Table 6. The FE specifications confirm that mobile money is associated with higher spending for all of these inputs, except for mineral fertilizer. The mobile money treatment effects are 4.1 thousand Ksh (50 US$) for hired labor, 2.5 thousand Ksh (30 US$) for organic fertilizer, and 1.2 thousand Ksh (15 US$) for chemical pesticides. These values are expressed per acre of banana production to have a comparable reference. It should be noted, however, that the majority of the sample households cultivate much less than one acre of banana. The average banana area per household is 0.37 acres.

**Table 6:** Determinants of input use in banana production.

	Hired labor		Organic fertilizer		Mineral fertilizer		Pesticides	
	(1)	(2)	(3)	(4)	(5)	(6)	(7)	(8)
Variable	FE	RE	FE	RE	FE	RE	FE	RE
Mobile money	4.122** (1.978)	0.810 (1.278)	2.502** (1.235)	1.267* (0.760)	−1.640 (1.147)	0.503 (0.737)	1.212* (0.628)	0.482 (0.403)
2010 dummy	5.706*** (1.111)	6.751*** (1.005)	2.471*** (0.694)	2.861*** (0.622)	6.118*** (0.644)	5.442*** (0.583)	2.388*** (0.353)	2.618*** (0.319)
Age		−3.0E-04 (0.043)		−0.024 (0.024)		−0.016 (0.024)		−0.029** (0.013)
Education		−0.017 (0.147)		−0.017 (0.086)		−0.051 (0.085)		−0.035 (0.047)
Male head		1.308 (1.390)		0.759 (0.813)		1.590** (0.809)		1.087** (0.442)
Household size		−0.230 (0.258)		−0.063 (0.150)		0.079 (0.150)		1.E-04 (0.082)
Land owned		−0. 004 (0.177)		0. 188* (0.104)		0.510*** (0.103)		0. 305*** (0.056)
Distance to market		−0.033 (0.143)		−0.112 (0.084)		0.058 (0.083)		−0.022 (0.046)
Distance to road		−0.010 (0. 136)		0.167** (0.079)		0.105 (0. 079)		0.054 (0. 043)
High-potential area		0.467 (1.034)		0.731 (0.604)		1.449** (0.602)		0.436 (0.329)
Intercept	−0.442	−1.595 (3.771)	−0.223	−0.587 (2.206)	1.846** (0.781)	−2.880 (2.194)	−0.460 (0.992)	−0.276 (1.198)
*Model statistics*								
Wald χ^2^		59.97***		48.34***		149.99***		133.91***
*F* value	31.20***		18.18***		56.23 ***		46.86***	
Hausman, χ^2^	4.76*		1.61		5.59*		2.29	

Notes: Estimates are based on balanced panel regressions with 640 observations and 320 groups. All dependent variables are measured in thousand Ksh per acre. Coefficient estimates can be interpreted as marginal effects; standard errors are shown in parentheses. FE, fixed effects; RE, random effects.

*,**,***significant at the 10%, 5%, and 1% level, respectively.

The RE specifications in Table 6 show that larger farms use more fertilizers and pesticides per acre. The same holds true for male-headed households, which is according to expectations. Female-headed households are often more constrained in their access to modern inputs and other productive resources. As some of the households do not use certain inputs, Tobit specifications of all input models are shown in Table S1. Most of the estimated coefficients increase in magnitude, suggesting that the linear model results are probably conservative estimates.

### Impact of mobile money on banana sales and profit

Results of the banana sales and profit models are shown in Table 7. Column (1) reveals that mobile money use is associated with a 10.4 percentage point higher proportion of banana sales (relative to total banana production). Given that non-users have sold 56% of their harvest in the market, the mobile money treatment effect implies a 19% increase in the degree of output commercialization. This confirms that mobile money services contribute to increased market transactions also on the output side. Unsurprisingly, sold proportions were higher in 2010, due to better rainfall and larger quantities harvested. The RE specification in column (2) further shows that larger farmers and those located in high-potential areas sell a larger proportion of their harvest. This is according to expectations, as these farmers also produce higher overall output.

**Table 7:** Determinants of banana sales and profits.

	Proportion of banana sales		Banana profits (thousand Ksh/acre)	
	(1)	(2)	(3)	(4)
Variable	FE	RE	FE	RE
Mobile money	0.104* (0.059)	0.084** (0.036)	30.112* (17.954)	17.486 (12.171)
2010 dummy	0.092*** (0.033)	0.098*** (0.030)	28.211*** (10.087)	32.004*** (9.198)
Age		−0.001 (0.001)		−0.258 (0.428)
Education		−0.002 (0.004)		−0.566 (1.467)
Male head		0.024 (0.038)		−5.307 (13.908)
Household size		0.001 (0.007)		−2.200 (2.384)
Land owned		0.014*** (0.005)		−3.657** (1.775)
Distance to market		2.5E-04 (0.001)		0.021 (0.301)
Distance to road		−0.003 (0.004)		−0.584 (1.341)
High-potential area		0.049* (0.028)		25.415** (10.259)
Intercept	0.537*** (0.040)	0.505*** (0.104)	7.901*** (12.233)	120.052*** (37.516)
*Model statistics*				
Wald χ^2^		40.60***		34.62***
*F* value	11.81***		11.62***	
Hausman test, χ^2^	0.17		0.20	

Notes: Estimates are based on balanced panel regressions with 640 observations and 320 groups. Coefficient estimates can be interpreted as marginal effects; standard errors are shown in parentheses. FE, fixed effects; RE, random effects.

*,**,***significant at the 10%, 5%, and 1% level, respectively.

The profit model results are shown in columns (3) and (4) of Table 7. The FE specification suggests that mobile money use contributes to higher banana profits in a magnitude of 30.1 thousand Ksh per acre (365 US$), implying a 35% gain over non-users. Impact pathways for profit gains may be through more intensive input use facilitated by mobile money and thus higher banana yields. Besides, reduced transaction costs in output markets may also play a role. The RE specification reveals that farmers in high-potential areas have higher profits. In contrast, profits per acre are somewhat lower on larger farms, indicating decreasing returns to scale in smallholder banana systems.

### Robustness checks

The results suggest that mobile money use contributes to increased household welfare through various pathways, including higher remittances received, higher farming intensity and profits, and a higher degree of commercialization. The estimated average treatment effect on household income of 61.5 thousand Ksh (equivalent to a 40% gain) is quite large. In this subsection, we carry out various additional tests to check how robust this treatment effect is to variations in the evaluation procedure.

One aspect that deserves more discussion is the potential effect of unobserved time-variant factors that might also influence household income. The FE panel specifications that we used in the impact models control for unobserved time-invariant heterogeneity, but not for time-variant factors. Unobserved time-variant factors do not cause a problem when they are uncorrelated with mobile money use. For instance, erratic weather conditions can affect farm household income, but we do not expect a systematic correlation with mobile money adoption (note that general location characteristics, such as soil and average rainfall conditions, are controlled for in the FE model). However, when unobserved factors that influence income are correlated with mobile money use, the treatment effect estimates would be biased. For instance, households that use mobile money may also adopt other technologies more rapidly. Mobile money adopters may also be more entrepreneurial, which could lead to higher efficiency in production and marketing beyond the effects of mobile money. Since the time period between our two survey rounds was only one year, the risk that time-variant heterogeneity causes a significant bias is small. Nonetheless, we test for such bias by using alternative specifications of the FE income model.

Results of these alternative specifications are shown in Table 8. Column (1) reproduces the original FE income model for comparison. In column (2), the same specification is used but this time excluding the non-adopters. Hence, the sample is confined to the early adopters of mobile money (those that adopted already in 2009) and the late adopters (those that adopted in 2010). Non-adopters differ significantly from the two adopter groups in terms of observed characteristics (Table S2), so one might expect that they also differ in terms of unobserved characteristics related to innovativeness and entrepreneurship. Yet, the results in Table 8 show that the treatment effect of mobile money does not change much when non-adopters are excluded from estimation. In columns (3) to (5) we use the full sample of farm households but additionally include time-variant factors, such as adoption of other technologies and market prices, as explanatory variables. We did not include these additional variables in the original model, because some of them may also be influenced by mobile money adoption. In column (3), we additionally include mobile phone ownership, in column (4) the adoption of tissue culture planting material for banana, and in column (5) individually reported prices for banana, mineral fertilizer, and pesticides. Some of these variables are significant, but the mobile money treatment effect remains robust. We cautiously conclude that the omission of time-variant factors does not lead to a significant bias.

**Table 8:** Treatment effects on household income with extended models.

	(1)	(2)	(3)	(4)	(5)
Variable	Original FE model	Non-adopters excluded, FE	Extended FE model	Extended FE model	Extended FE model
Mobile money	61.47* (32.70)	68.58** (33.96)	69.55** (34.23)	63.87* (32.65)	61.61* (32.31)
2010 dummy	73.34*** (18.37)	66.23*** (19.97)	74.27*** (18.42)	69.11*** (18.50)	−10.57 (33.10)
Mobile phone ownership			−50.49 (62.88)		
Tissue culture adoption				185.24* (111.86)	
Banana price					2.35 (1.99)
Fertilizer price					1.58** (0.65)
Pesticide price					0.06 (0.04)
Intercept	168.31*** (22.28)	170.23*** (24.98)	206.87*** (52.96)	58.05 (70.19)	124.00*** (33.85)
*Model statistics*					
*F* value	20.38***	17.55***	13.79***	14.58***	10.58***
Number of observations	640	584	640	640	640

Notes: The dependent variable in all models is total household income measured in thousand Ksh/year. Coefficient estimates can be interpreted as marginal effects; standard errors are shown in parentheses. FE, fixed effects.

*,**,***significant at the 10%, 5%, and 1% level, respectively.

**Figure 3:**
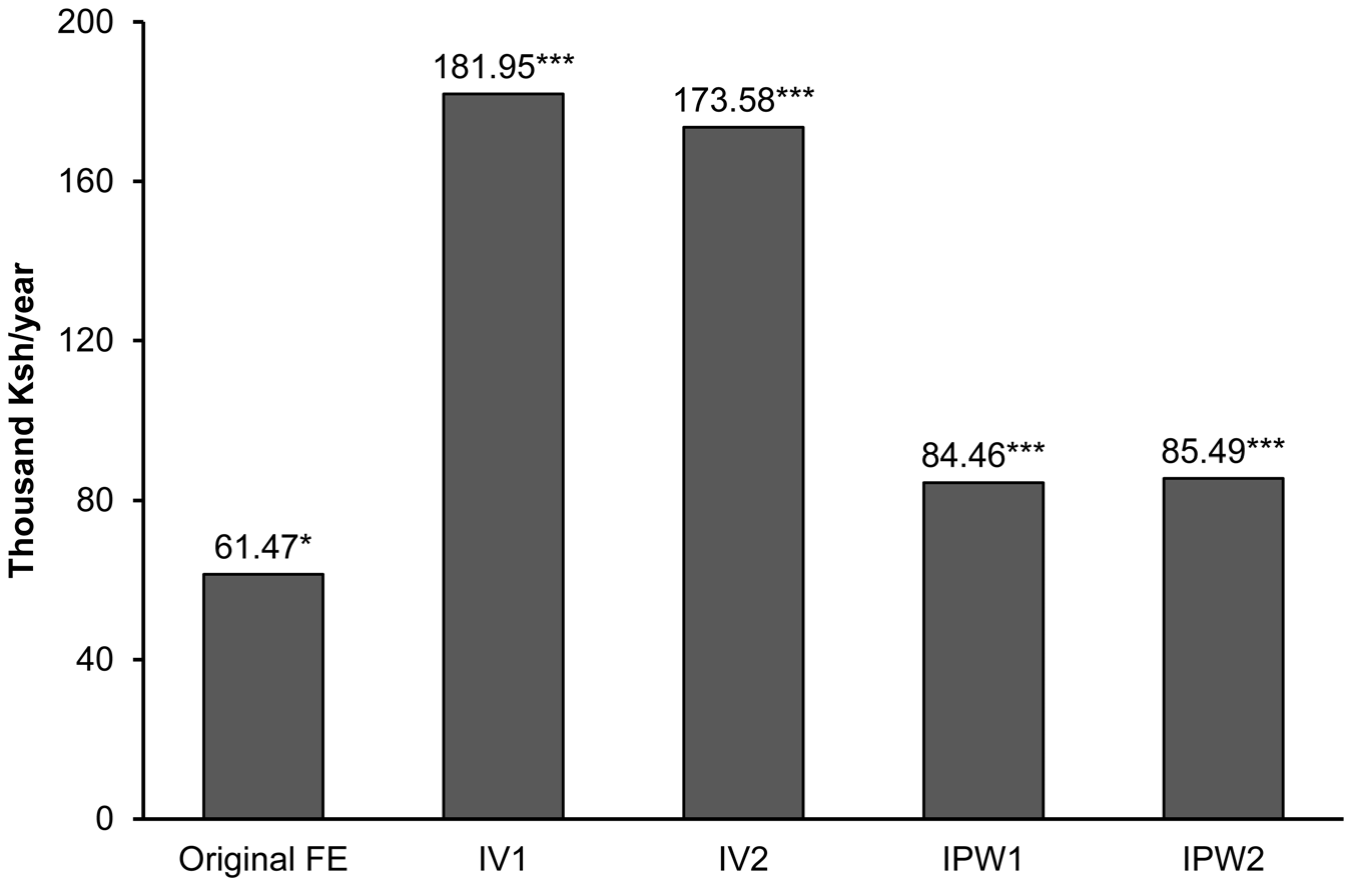
Treatment effects on household income with alternative estimators. Notes: Original FE refers to the fixed effects model shown in Table 4, column (1). IV1 is based on an instrumental variable estimator where mobile money was instrumented with the percentage of households using mobile money at the village level. IV2 is based on an instrumental variable estimator where mobile money was instrumented with the percentage of households owning a mobile phone at the village level. IPW1 is based on an inverse probability estimator where the original probit model shown in Table 3, column (1), was used to calculate propensity scores. IPW2 is based on an inverse probability estimator where the original probit model was extended by variables measuring prices of banana, fertilizer, and pesticides. *,*** significant at the 10% and 1% level, respectively.

Furthermore, we used instrumental variable (IV) and inverse probability weighting (IPW) estimators as robustness checks. Both are common tools in the impact assessment literature to reduce bias resulting from unobserved and observed heterogeneity [39]. In the IV regressions, we instrumented mobile money use of individual households with the proportion of households using mobile money and owning a mobile phone at the village level. These instruments are correlated with mobile money use and do not affect individual income directly. Interestingly, the IV treatment effects on income are even larger than those from the original model (Figure 3). These IV results should not be overinterpreted, because the instruments used may not be completely exogenous. For the IPW models, we estimated propensity scores in a first step, which were then used to calculate analytic weights for the second stage regression. The results suggest that the original mobile money treatment effect may actually be a conservative estimate (Figure 3).

## Conclusion

Previous research had documented the rapid spread of mobile phone based money services in developing countries. Existing studies also suggest that this may have positive effects especially for poor people in rural areas who are often underserved by the traditional banking system. In this article, we have contributed to the literature by analyzing the impact of mobile money use on the income of smallholder farm households, which has never been done previously. Furthermore, we have examined possible impact pathways by looking at the influence of mobile money on remittances received, transactions in agricultural input and output markets, and farm profits. The empirical analysis has concentrated on banana-growing households in Kenya, where mobile money services have spread rapidly in recent years. Panel survey data were collected and used for this analysis.

Results show that mobile money use has a positive net impact on household income. One important impact pathway is through remittances received, which are much higher for users of mobile money. In comparison to traditional formal and informal mechanisms of transferring money between relatives and friends, mobile money services reduce the transaction costs substantially. These services also provide new incentives for saving. And, mobile money contributes to more commercially-oriented farming. Our results reveal that mobile money users apply significantly more purchased inputs – such as fertilizer, pesticides, and hired labor – and sell a larger proportion of their harvest in the market. On the one hand, this is related to lower transaction costs in terms of paying and receiving money from business partners. On the other hand, more remittances and savings seem to reduce risk and liquidity constraints. Mobile money users have 35% higher profits per acre of banana production.

Our results confirm that mobile money services can be welfare-enhancing for smallholder farm households, who constitute the majority of the rural poor. In Kenya, mobile money also seems to be widely accessible. While wealthier and better educated households were among the first to adopt this innovation, within only a few years more than 90% of all households in our sample were using mobile money services. Mobile money can help to overcome some of the important market access constraints of smallholder farm households. It is noteworthy to stress that the rapid spread of mobile services in Kenya is entirely driven by private sector incentives, underlining that the private sector has an important role to play for rural development. Through sensible regulations, the public sector needs to ensure that the emerging markets are competitive.

Our study has focused on banana growers in two provinces of Kenya, so the concrete numerical results should not be generalized widely. In spite of various robustness checks that we carried out, we also acknowledge that it is difficult to eliminate all potential biases in impact evaluation when building on observational data. Follow-up research should analyze the access to mobile money and the wider implications under diverse conditions to gain a more comprehensive picture of potentials and limitations. Also the analysis of impact pathways and broader social ramifications deserves further attention. One interesting question is how mobile money services affect informal savings and insurance mechanisms at the local level.

## Supporting Information

Table S1 Determinants of input use in banana production (Tobit estimates).
(PDF)

Table S2 Characteristics of early, late, and non-adopters of mobile money.
(PDF)

Data S1 (DTA)
