# Supplementary material for: Mobile Money, Smallholder Farmers, and Household Welfare in Kenya
Source: PLoS One. 2014 Oct 6;9(10):e109804. doi: 10.1371/journal.pone.0109804 (PMC4186858; doi:10.1371/journal.pone.0109804)
Supplement: Table S1 — Determinants of input use in banana production (Tobit estimates). (PDF) [file pone.0109804.s001.pdf]

Supporting information

Table S1. Determinants of input use in banana production (Tobit estimates)

Variable	Hired labor	Organic fertilizer	Mineral fertilizer	Pesticides
Mobile money	3.625 (2.373)	3.881*** (1.583)	0.814 (1.473)	2.373** (1.121)
2010 dummy	12.751*** (1.891)	14.975*** (1.329)	17.346*** (1.295)	10.854*** (0.941)
Age	-0.011 (0.074)	-0.073* (0.044)	-0.031 (0.043)	-0.068 (0.029)
Education	0.101 (0.252)	-0.009 (0.150)	-6.0E-04 (0.143)	-0.017 (0.100)
Male head	0.337 (2.385)	1.413 (1.447)	2.523* (1.398)	3.083*** (1.020)
Household size	-0.486 (0.449)	-0.027 (0.266)	0.127 (0.260)	0.030 (0.179)
Land owned	0.500* (0.296)	0.479*** (0.172)	0.895*** (0.165)	0.659*** (0.112)
Distance to market	0.090 (0.243)	-0.123 (0.145)	0.130 (0.14	-0.006 (0.096)
Distance to road	-0.264 (0. 241)	0.211 (0. 136)	0.105 (0. 134)	0.147 (0. 090)
High-potential area	2.833 (1.779)	2.137** (1.067)	4.290*** (1.044)	2.153*** (0.719)
Intercept	-13.630** (6.594)	-14.190*** (4.074)	-19.651*** (3.930)	-14.130*** (2.781)
Model statistics				
Wald χ^2	72.75***	245.74***	317.30***	285.55***
Log likelihood	-3763.74	-3671.48	-3553.05	-2625.99

*,**,*** significant at the 10%, 5%, and 1% level, respectively.
