# Supplementary material for: Mobile Money, Smallholder Farmers, and Household Welfare in Kenya
Source: PLoS One. 2014 Oct 6;9(10):e109804. doi: 10.1371/journal.pone.0109804 (PMC4186858; doi:10.1371/journal.pone.0109804)
Supplement: Table S2 — Characteristics of early, late, and non-adopters of mobile money. (PDF) [file pone.0109804.s002.pdf]

Supporting information

Table S2. Characteristics of early, late, and non-adopters of mobile money

Variable	Early adopters (adopted in 2009)	Late adopters (adopted in 2010)	Non-adopters (not adopted until 2010)
Land owned (acres)	3.50 (2.86)	3.18 (3.31)	2.86* (2.64)
Age of household head (years)	58.45 (13.43)	56.98 (12.76)	68.36*** (14.49)
Education (years)	9.21 (3.95)	8.15*** (3.48)	4.30*** (3.96)
Household size (members)	4.75 (1.97)	4.55 (2.09)	3.58*** (1.69)
Male household head (dummy)	0.85 (0.36)	0.82 (0.38)	0.68*** (0.47)

Notes: Mean values are shown with standard deviations in parentheses. Mean value differences were tested for late adopters and non-adopters in comparison to early adopters. *,*** differences are significant at the 10% and 1% level, respectively.
